# Oxidative Stress Induced by Metal Ions in Bioleaching of LiCoO_2_ by an Acidophilic Microbial Consortium

**DOI:** 10.3389/fmicb.2019.03058

**Published:** 2020-01-15

**Authors:** Xiaocui Liu, Hao Liu, Weijin Wu, Xu Zhang, Tingyue Gu, Minglong Zhu, Wensong Tan

**Affiliations:** ^1^State Key Laboratory of Bioreactor Engineering, East China University of Science and Technology, Shanghai, China; ^2^Department of Chemical and Biomolecular Engineering, Ohio University, Athens, OH, United States

**Keywords:** waste lithium ion battery, bioleaching, ROS, glutathion, acidophilic microbial consortium, biofilm

## Abstract

An acidophilic microbial consortium (AMC) was used to investigate the fundamental mechanism behind the adverse effects of pulp density increase in the bioleaching of waste lithium ion batteries (WLIBs). Results showed that there existed the effect of metal-ion stress on the bio-oxidative activity of AMC. The Li^+^ and Co^2+^ accumulated in the leachate were the direct cause for the decrease in lithium and cobalt recovery yields under a high pulp density. In a simulated bioleaching system with 4.0% (w ⋅v^–1^) LiCoO_2_, the intracellular reactive oxygen species (ROS) content in AMC increased from 0.82 to 6.02 within 24 h, which was almost three times higher than that of the control (2.04). After the supplementation of 0.30 g⋅L^–1^ of exogenous glutathione (GSH), the bacterial intracellular ROS content decreased by 40% within 24 h and the activities of intracellular ROS scavenging enzymes, including glutathione peroxidase (GSH-Px) and catalase (CAT), were 1.4- and 2.0-folds higher in comparison with the control within 24 h. In the biofilms formed on pyrite in the bioleaching of WLIBs, it was found that metal-ion stress had a great influence on the 3-D structure and the amount of biomass of the biofilms. After the exogenous addition of GSH, the structure and the amount of biomass of the biofilms were restored to some extent. Eventually, through ROS regulation by the exogenous addition of GSH, very high metal recovery yields of 98.1% Li and 96.3% Co were obtained at 5.0% pulp density.

## Introduction

Bioleaching has been used to recover metals from mineral ores (Sun et al., 2010), waste printed circuit boards (PCBs) (Yang et al., 2014), contaminated river sludge (Zhang et al., 2018), fly ash (Margallo et al., 2015), and spent catalysts with the advantages of low cost, mild reaction conditions, and environmental friendliness. The increased uses of lithium ion batteries in electric cars and electronic devices have led to accumulations of large amounts of waste lithium ion batteries (WLIBs). Bioleaching has emerged as a promising technology in metal recovery from WLIBs (Li et al., 2013).

The bioleaching mechanism of recovering Li and Co from WLIBs can be illustrated by Reactions (1) to (4) using pyrite as the energy source for acidophilic bacteria.

(1) $$2\,F\,e^{2+}+2\,H^{+}+0.5\,O_{2}\to 2\,F\,e^{3+}+H_{2}\,O\,\left(\mathrm{microbial}\,\mathrm{action}\right)$$

(2) $$F\,e\,S_{2}+2\,F\,e^{3+}\to 3\,F\,e^{2+}+2\,S$$

(3) $$2\,S+3\,O_{2}+2\,H_{2}\,O\to 2\,H_{2}\,S\,O_{4}\,\left(\mathrm{microbial}\,\mathrm{action}\right)$$

(4) $$2\,L\,i\,C\,o\,O_{2}+8\,H^{+}+2\,F\,e^{2+}\to 2\,L\,i^{+}+2\,C\,o^{2+}+2\,F\,e^{3+}+4\,H_{2}\,O$$

In Reaction 1, *Acidithiobacillus ferrooxidans*, which is an iron-oxidizing bacterium (IOB) uses Fe^2+^ as electron donor (energy source) for oxygen respiration (Gu and Wong, 2004; Quatrini et al., 2009). FeS_2_ is the original electron donor source in this system. Fe^3+^ reacts with FeS_2_ to gain one electron and becomes Fe^2+^ in Reaction 2. Fe^2+^ passes the electron to a redox active protein, such as rusticyanin on the cell’s outer membrane, and it converts Fe^2+^ back to Fe^3+^. Thus, Fe^3+^/Fe^2+^ acts as an electron shuttle for the extracellular electron transfer (EET) (He et al., 2017; Liu et al., 2018). Furthermore, the elemental sulfur released by FeS_2_ also serves as an electron donor for oxygen respiration by sulfur-oxidizing bacteria such as *A. ferrooxidans* as shown in Reaction 3. In this case, S^0^ diffuses into the cell’s cytoplasm for oxidation to release electrons for oxygen respiration (Yin et al., 2014). This process produces proton (oxidant) that biosolubilizes Li from LiCoO_2_ with the help of Fe^2+^ as electron donor as shown in Reaction 4. At the same time, Co^3+^ in LiCoO_2_ is also reduced to soluble Co^2+^ by Fe^2+^.

Despite the various advantages of bioleaching, it suffers from disadvantages such as longer bioleaching cycle, lower leaching efficiency, and lower pulp density, which limit bioleaching’s practical applications on an industrial scale. The decline of the efficiency of bioleaching of valuable metals from WLIBs at high pulp densities leads to small solid waste loading or larger equipment size.

Bioleaching efficiency can be affected by many factors. Besides bacterial species and energy sources (Sugio et al., 1989; Bajestani et al., 2014), factors such as pH (Li et al., 2013), dissolved oxygen (DO), dissolved CO_2_ (Wang et al., 2015), mineral/solid waste particle size (Jones et al., 2011), and metal ion concentration (e.g., Ni^2+^, V^4+^, Mo^6+^) (Pradhan et al., 2009) have also been investigated by researchers. It was found that a higher bioleaching capacity could be obtained from mixed cultures of some acidophilic bacteria (Ilyas and Lee, 2015) compared with using pure-strain bacteria, owing to synergistic effects. Nonetheless, the pulp density of cathode active material is still quite low in the bioleaching of WLIBs, although several optimization strategies have been developed. For example, Niu et al. (2014) investigated the bioleaching of WLIBs under different pulp densities. They found that Li recovery yields decreased greatly under the pulp density of 4.0% (w⋅v^–1^) compared to that under 1.0%. Similarly, results from the bioleaching of waste Zn–Mn batteries also showed that when the pulp density was increased from 1.0 to 8.0%, the recovery yields of Zn and Mn decreased from 100.0 and 94.0% to 29.9 and 2.5%, respectively (Xin et al., 2012).

When bioleaching of valuable metals from WLIBs is carried out using acidophilic microbial consortium (AMC), the concentrations of the metal ions in the leachate, such as Li^+^ and Co^2+^, would increase with the increase of pulp density. It has been found that high concentrations of metal ions may lead to the death of bacteria because of the cytotoxicity of the metal ions (Leduc et al., 1997), high osmotic pressure stress (Watling et al., 2016), high oxidative stress (Adam et al., 2012), etc. Oxidative stress in acidophilic bacteria could also occur in the bioleaching system due to extremely acidic environments (Ferrer et al., 2016). In bacterial cells, a reactive oxygen species (ROS) is a signaling molecule for the activation of the defense mechanisms inside bacterial cells (Gilroy et al., 2014). However, a large increase of the intracellular ROS content will result in the peroxidation of lipid membrane accumulation of malonyldialdehyde (MDA) (Dat et al., 2000), damage of DNA, and the reduction of the caspase-3 enzyme activity (Kim et al., 1995). It is found that the increase of the intracellular ROS content resulted from Cu^2+^ accumulation can lead to the death of *A. ferrooxidans* (Xia et al., 2011). Fortunately, it has been proven that an intracellular ROS scavenging system exists in *Leptospirillum ferrooxidans* to improve the damage resistance (Javiera et al., 2012). Exogenous cobalamin has also been used to decrease the ROS content in *L. ferrooxidans*, and to improve cell growth and survival (Ferrer et al., 2016). Researchers also have found some ROS scavenging enzymes in acidophilic microorganisms, including glutathione peroxidase (GSH-Px), catalase (CAT), and superoxide dismutase (SOD) (Maaty et al., 2009). This suggests that it is possible to use different ROS scavengers to regulate intracellular ROS levels. Therefore, it is necessary to study the effect of intracellular ROS induced by metal ions under a high pulp density on the activities of AMC and the metal recovery yields of lithium and cobalt in the bioleaching of WLIBs. However, the mechanism of the influence of ROS induced by Li^+^ and Co^2+^ in AMC under a high pulp density of LiCoO_2_ has not been reported so far.

Researchers have started to realize that biofilm formation has a very important role in bacterial activities during bioleaching in natural environments and industrial settings (González et al., 2013). The sessile cell volumetric densities are easily 10^2^ or 10^3^ higher than that of planktonic cells, which means the chemical concentrations underneath a biofilm are much higher than those in the bulk-fluid phase. However, biofilms formed on pyrite in bioleaching of WLIBs, the effects of oxidative stress induced by Li and Co ions on biofilm formation have yet to be reported.

In this work, an AMC containing mainly *Leptospirillum ferriphilum* and *Sulfobacillus thermosulfidooxidans* was used to study bioleaching of WLIBs. LiCoO_2_ is a cathode active material in WLIBs. The main objective was to find the relationships among LiCoO_2_ pulp density, metal concentrations, intracellular ROS content, activities of ROS scavenging enzymes, biofilm morphology, and metal recovery yields. Furthermore, the addition of an effective ROS regulator was explored to decrease the intracellular ROS content induced by metal ions. A fundamental mechanism and an effective strategy to improve the lithium and cobalt recovery yields from bioleaching of WLIBs under a high pulp density were proposed.

## Materials and Methods

### Materials and Bacteria

A pyrite sample was kindly donated by a mining company in China. It consisted mainly of (30.0 ± 0.5)% (w⋅w^–1^) Fe, (28.0 ± 0.3)% (w⋅w^–1^) S, and (3.0 ± 0.1)% (w⋅w^–1^) As. The pyrite sample was processed using following steps: washing with water and a 20% (v⋅v^–1^) H_2_SO_4_ aqueous solution successively, drying, grinding, and sieving to obtain 63–90 μm particles. LiCoO_2_ powder was purchased from Adamas Reagent Co., Ltd., in Shanghai, China. It had a purity of 99.8% and particle size 105–130 μm.

**FIGURE 1:**
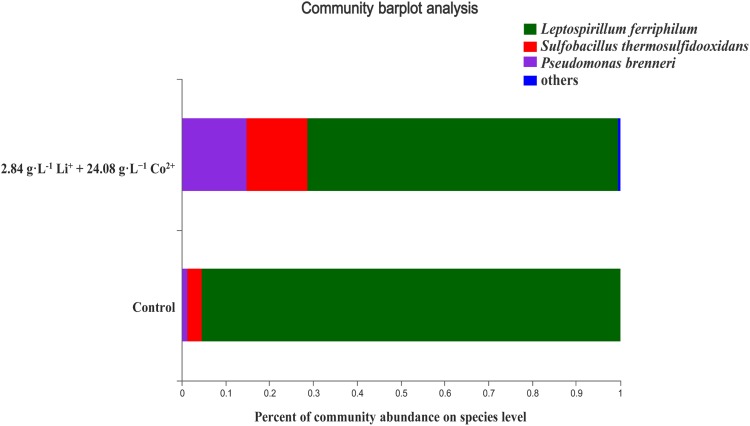
The structural composition of AMC under the control and test conditions.

Acidophilic microbial consortium used in this study mainly contained *L. ferriphilum* and *S. thermosulfidooxidans*. The AMC was initially isolated from an acid mine drainage and had gone through a long process of selection and domestication. The structural composition of AMC under control and test condition is shown in Figure 1. Under the test condition, the structure of the consortium did not change. Although the microbial population of the mixed consortium changed under the test condition, *L. ferriphilum* remained the dominant bacterial species. It was sequenced for the V4 region of 16S rRNA using a high-throughput RNA sequencing technique (Wu et al., 2018). 9K medium was used. Its composition was: (NH_4_)_2_SO_4_ 3.0 g⋅L^–1^, KCl 0.1 g⋅L^–1^, MgSO_4_⋅7H_2_O 0.5 g⋅L^–1^, K_2_HPO_4_ 0.5 g⋅L^–1^, and Ca (NO_3_)_2_ 0.01 g⋅L^–1^. Based on the results of previous studies of our laboratory, 10.0% of the pyrite powder was added as an iron source and energy source. The culture medium’s initial pH was adjusted to 1.25 using sulfuric acid before inoculation.

### Bioleaching Lithium and Cobalt From LiCoO_2_ Under Different Pulp Densities

A two-step method was adopted, in which AMC was pre-cultured in a pyrite slurry to improve its bacterial activity, and LiCoO_2_ was then added to initiate the bioleaching process with exogenous-acid adjustment (Xin et al., 2012). As the ORP value was an indicator of bacterial oxidation activity, it was stipulated that a series of exploratory experiments should be carried out in the cultivation system under the ORP value of 530 mV. Each 250 mL flask contained 90 mL sterilized 9K medium, 10 g of sterilized pyrite particles and 10 mL inoculum. The flasks were incubated in an orbital shaker incubator (TQHZ-2002A, Taicang, Jiangsu, China) at 42°C and 180 r/min for 48 h. When the ORP value of the slurry reached 530 mV, the LiCoO_2_ powder was added into different flasks at pulp densities of 3.0, 4.0, and 5.0%, respectively, while 0% was used as the control. During the bioleaching process, to maintain the pH stability of the whole bioleaching system and to ensure that AMC could survive in the suitable growth conditions, the pH of the slurry was maintained at the initial value of 1.25 through sulfuric acid addition every 2 h. pH, ORP, the Fe^3+^ generation rate, concentration of total Fe ions, Fe^2+^, Li^+^, and Co^2+^ concentrations were monitored every day.

### Effect of Different Concentrations of Li^+^, Co^2+^ on Bio-Oxidative Activity of AMC

In order to study the tolerance of AMC to Li^+^ and Co^2+^, several simulated bioleaching systems were adopted. When the ORP of the pulp slurry reached 530 mV, different amounts of Li_2_SO_4_ were dissolved in the pyrite slurry to form simulated solutions with final Li^+^ concentrations of 5.0, 6.0, and 7.0 g⋅L^–1^, respectively. Similarly, 50.0, 60.0, and 70.0 g⋅L^–1^ of Co^2+^ simulated solutions were made by using CoSO_4_⋅7H_2_O to investigate the tolerance of AMC to Co^2+^. To simulate the ionic environment upon the complete bioleaching of LiCoO_2_, different amounts of Li_2_SO_4_ and CoSO_4_⋅7H_2_O were simultaneously dissolved in the pyrite slurry. The final concentrations of Li^+^ and Co^2+^ in the slurry were equivalent to those after complete bioleaching of LiCoO_2_ at the pulp densities of 3.0, 4.0, and 5.0%, respectively. For example, the concentration of Li^+^ was 2.1 g⋅L^–1^ when 3% LiCoO_2_ was completely dissolved. Based on the concentration of Li^+^, the corresponding Li_2_SO_4_ concentration was 16.6 g⋅L^–1^. These systems were labeled as the simulated bioleaching systems for short in this work. pH, ORP, Fe^3+^ generation rate, and Fe^2+^ concentration were monitored every day. The control tests were performed by bioleaching 10.0% pyrite pulp without any Li^+^ and Co^2+^. All the tests were carried out in triplicates.

### Detection of Intracellular ROS Content in the Presence of Li^+^ and Co^2+^

The bacterial intracellular ROS content in the simulated bioleaching systems at different time was detected using an ROS assay kit (Farrell et al., 2011) following a reported experimental method (Wang et al., 2015). Five milliliters of slurry was centrifuged at 450 *g* for 10 min to harvest free cells and pyrite particles. The sessile bacteria adhering to the pyrite particles in each flask were dislodged by adding an isometric Tween-20 liquid and then shaken in an oscillator (WH-861, Shanghai Zuoyan Instrument Technology Co., Ltd., Shanghai, China) for 3 min until the bacterial cell count in the supernatant counted under a microscope stopped increasing. The Tween-20 liquid used here was 0.1% Tween-20 dissolved in 9K medium at pH 1.25. After shaking, the supernatant was harvested. This process was repeated twice. The bacterial suspension including all of the planktonic and sessile bacteria was centrifuged at 450 *g* for 10 min, and then the cell pellet was re-suspended in 5 mL sterile 9K medium. One microliter of 2’,7’-dichlorofluorescin diacetate (DCFH-DA) probe was then added to 1 mL of the cell re-suspension solution. After that, the cell suspension was kept away from light at 37°C for 20 min in a water bath. After centrifugation at 16,000 *g* for 2 min, the cell pellet was washed with 1 mL of sterile water. Then, this washing and centrifuging process was repeated three more times.

The cell pellet was re-suspended again in 200 μL of sterile water and then shaken well and transferred to a non-transparent 96-well plate. Then, it was read under a micro-plate reader with excitation and emission wavelengths of 485 and 535 nm, respectively. The fluorescence (FL) value is positively correlated with the ROS content. OD_600_ (optical density at a wavelength of 600 nm) was used to represent the amount of cells in a re-suspension solution. The ratio of the FL value to the OD_600_ (FL/OD_600_) was defined as the intracellular ROS content per unit cell. The FL/OD_600_ of the cell re-suspension solution without Li^+^ and Co^2+^ was used as the standard of intracellular ROS content (Farrell et al., 2011). The test was run in triplicate.

### The Effect of Exogenous Glutathione (GSH) on Bacterial Growth, Intracellular ROS Content, and Activities of Intracellular ROS Scavenging Enzymes

The simulated bioleaching system with 4.0% pulp density of LiCoO_2_ was used to investigate the effects of exogenous glutathione (GSH) on bioleaching characteristics and intracellular ROS content. The two-step method and the exogenous-acid adjustment technique were also adopted. After 12 h cultivation, 0.03 g GSH powder (in reduced form, Shanghai Aladdin Biochemical Technology Co., Ltd., Shanghai, China) was added into the slurry to reach 0.30 g⋅L^–1^. The flask was shaken slightly until the added GSH was dissolved. The slurry pH, ORP, Fe^3+^ generation rate, Fe^2+^ concentration, intracellular ROS content, and bioleaching efficiency of lithium and cobalt were monitored every 12 h.

Activities of intracellular ROS scavenging enzymes in the simulated bioleaching system with 4.0% pulp density were tested to verify GSH regulation of intracellular ROS content. The improved 9K medium, in which energy source was replaced by 44.2 g⋅L^–1^ of FeSO_4_⋅7H_2_O and 0.2 g⋅L^–1^ of yeast extract, was applied here to cultivate AMC. GSH was added into the medium to reach 0.3 g⋅L^–1^ after 12 h cultivation. Twelve hours after the addition of GSH, 320 mL of the leachate was taken, and then centrifuged at 11,000 *g* for 10 min at 4°C to harvest bacterial cells. The harvested cells were then re-suspended in a 10 mL buffer solution (30 mM of NaCl and 30 mM of Tris-HCl, pH 8.0) to be lyzed using ultrasound. The ultrasound conditions were: cells broken for 4 s and then cooled for 4 s, continued for 1 min, and then repeated 15 times at 20 s intervals. The cell lysate was centrifuged after the ultrasonic treatment at 17,500 *g* for 10 min. Then, the supernatant was stored in a freezer at −20°C for the subsequent enzyme activity assays. Both the enzyme activity assays and the enzyme activity definitions for GSH-Px, CAT, and SOD were as described in the literature (Liang et al., 2014). In these tests, the controls were the corresponding simulated bioleaching systems without GSH addition. All the tests were carried out in triplicate.

### Analytical Determination

pH and ORP were measured using a pH/ORP meter (Mettler model FE20, Shanghai, China). The Fe^2+^ concentration was measured using the dichromate titration method (Sun et al., 2012), which was based on the redox reaction between Fe^3+^ and elemental copper. The reaction was shown in Eq. 5 (Arshadi and Mousavi, 2014):

(5) $$2\,F\,e^{3+}+C\,u\to 2\,F\,e^{2+}+C\,u^{2+}$$

Total Fe ion concentration was obtained by adding excessive copper powder to a leachate sample, and the Fe^3+^ concentration was equal to total Fe ion concentration minus the Fe^2+^ concentration. The Li^+^ and Co^2+^ concentrations were measured using an atomic absorption spectrophotometer (iCE 3000 SERIES AA, Great Britain). The ROS assay kit (catalog no. S0033) was purchased from Biyuntian Biological Reagent Co., Ltd., Shanghai, China. FL was obtained using a micro-plate reader (VARIOSKAN LUX, Finland). OD_600_ was determined on an ultraviolet–visible spectrophotometer (UV-2102C, Unico Shanghai Instruments Co., Ltd., Shanghai, China). Bioleaching tests were carried out in flasks placed in an orbital shaker incubator (TQHZ-2002A, Taicang, Humeri Biochemical Instrument Factory, Interchange, China) at 42°C and 180 r/min.

The Fe^3+^ generation rate (*V*) and metal recovery yield (*Y*) of lithium and cobalt were calculated from the following equations:

(6) $$V=\frac{C_{2}-C_{1}}{t_{2}-t_{1}}$$

(7) $$Y=\frac{C}{X\times 10\times M\times 100}\times 100\%$$

Where *C*_1_ and *C*_2_ are Fe^3+^ concentrations in the leaching solution at time *t*_1_ and time *t*_2_, respectively. *C* is Li^+^ or Co^2+^ concentration (mg⋅L^–1^), *X* the pulp density of LiCoO_2_, and *M* the mass fraction of element Li or Co in LiCoO_2_.

### Staining and Imaging of Bio Films

L13152 LIVE/DEAD^®^ Backlight Bacterial Viability Kit (Molecular Probes, Sigma, Shanghai), which includes green-fluorescent stain SYTO 9 and red-fluorescent stain presidium iodide (PI), was used to stain biofilms (Guo et al., 2018). SYTO9 penetrates all cell membranes, while PI only penetrates damaged cell membranes. Thus, living cells show up in green color (excitation wavelength 488 nm), while dead cells appear red (wavelength 561 nm).

Flaky pyrite coupons with dimensions of 1 cm × 1 cm × 0.5 cm were polished sequentially with 180, 400, and 600 grit sandpapers. They were then sterilized at 121°C for 20 min. The pyrite coupons were equally divided into five groups and placed in five shake flasks. Ninety liters of 9K medium and 10 L of bacterial suspension inoculate were added to each shake flask and then incubated at 42°C. The moisture lost due to evaporation was replenished every 24 h. When the E_*h*_ (red ox potential) value of the slurry reached 550 V after 3 days of cultivation, bio films were found to have formed on the surface of the pyrite. At this time, 2.23 g of Li_2_SO_4_ was added to the first group, and 11.47 g of CoSO_4_⋅7H_2_O was added to the second group. Both 2.23 g of Li_2_SO_4_ and 11.47 g CoSO_4_⋅7H_2_O were added to the third and the fourth groups. None was added to the fifth group (control group). Five experimental groups were subjected to static incubation at 42°C. After 12 h of incubation, 0.03 g of reduced agglutination (GSH) was added to the fourth group.

The bio film staining process was performed as follows: The pyrite coupons were retrieved from flasks after 7 days incubation after the bio films formed on it. They were gently rinsed with 9K medium. The staining solution was then dropped onto bio films on the coupon surfaces and allowed to sit at room temperature for 15 min in the dark. The excess staining solution was washed away with antagonized water, and the coupons were observed using a confocal laser scanning microscopy (CLSM) (Fluoview FV300, Olympus Optical, Tokyo, Japan) at 40× magnification.

### Sessile Cell Count

Five grams of pyrite particles with an average diameter of 75 μM was added to three 250 L shake flasks. Then, 90 L 9K medium and 10 L bacterial suspension (inoculate) were added to the flasks before incubation at 42°C in an incubator (180 r/min). After 2 days, mature bio films formed. Then, 2.23 g Li_2_SO_4_ and 11.47 g CoSO_4_⋅7H_2_O were added into one of the shake flasks. Another shake flask was added 2.23 g Li_2_SO_4_, 11.47 g CoSO_4_⋅7H_2_O, and 0.03 g GSH. The last one without Li_2_SO_4_, or CoSO_4_⋅7H_2_O, GSH addition served as control.

After 4 days of incubation, the pyrite particles in shake flask was taken out and washed with 9K medium to remove planktonic bacteria on the surface of the particles. Then, the particles were added to a 9K medium containing 0.1% Tween-20 and shaken for 5 min to wash away sessile cells. The slurry was then centrifuged at 450 *g* for 1 min to collect the supernatant, and the number of sessile cells was enumerated with a hemocytometer at 400× magnification under a light microscope. According to the number of sessile cells and the total weight, average diameter, and the density of pyrite particles, the number of sessile cells in specific surface area of pyrite particles was calculated.

## Results and Discussion

### Impact of Pulp Density on AMC Activity and Bioleaching Efficiency

To maintain the activity of AMC during bioleaching, a two-step method was used for the bioleaching of LiCoO_2_ from WLIBs. The AMC growth curve was determined experimentally. The total number of bacteria, including planktonic and sessile bacteria in the bioleaching system, was obtained through the Tween-20 method (section “Detection of Intracellular ROS Content in the Presence of Li^+^ and Co^2+^”). LiCoO_2_ was added to the slurry for bioleaching at the late stage of the exponential growth phase of AMC.

According to the literature (Niu et al., 2014), the stability of pH is very important for AMC to maintain their high bioleaching activities. The changes of pH value with time were determined experimentally during the exogenous-acid adjustment process under 5.0% pulp density of LiCoO_2_. It was demonstrated that the pH of the leaching solution rose rapidly when LiCoO_2_ was added to the 9K medium. Thus, the slurry pH had to be adjusted back to 1.25 every 2 h using sulfuric acid. It was an acid consumption process for the bioleaching of LiCoO_2_ under pulp density of 5.0% in the first 12 h. The pH of the pulp slurry became relatively stable without sulfuric acid addition after 12 h.

**FIGURE 2:**
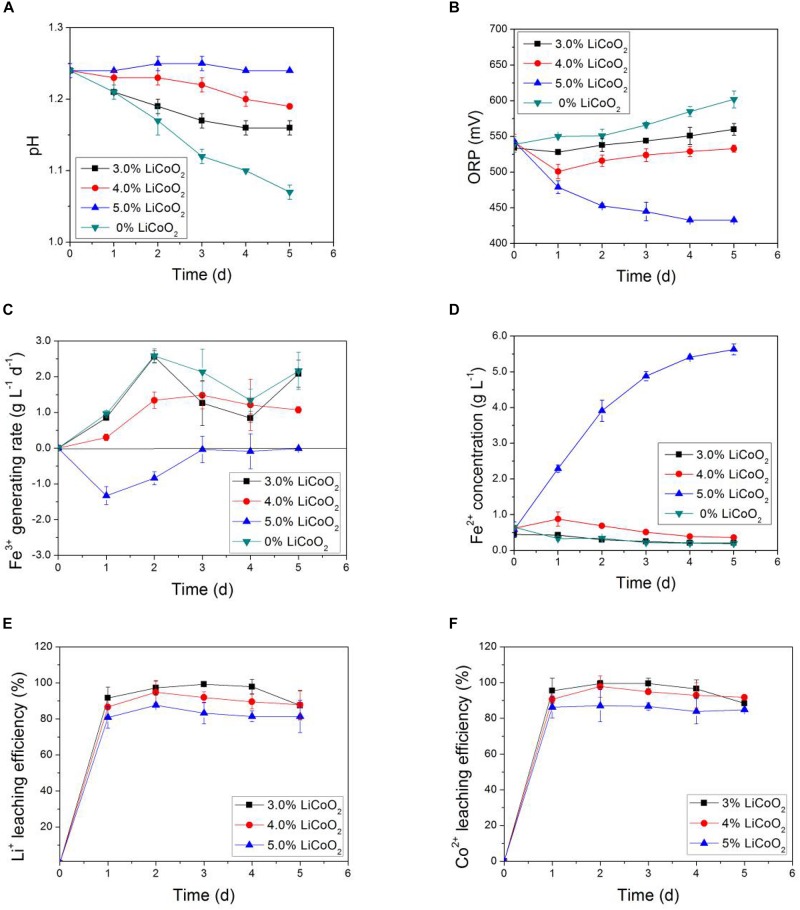
Time-courses for **(A)** pH, **(B)** ORP, **(C)** Fe^3+^ generation rate, **(D)** Fe^2+^ concentration, **(E)** bioleaching efficiency of lithium, and **(F)** bioleaching efficiency of cobalt during bioleaching process of LiCoO_2_ using the two-step method and the exogenous-acid adjustment technique.

Through the two-step method and the exogenous-acid adjustment technique, the variations of relevant parameters, including pH, ORP, Fe^3+^ generation rate, Fe^2+^ concentration, and bioleaching efficiencies of lithium and cobalt from LiCoO_2_ as a function of time under different pulp densities were obtained. They are shown in Figure 2. The pH was related to the sulfur-oxidizing ability of sulfur-oxidizing bacteria in AMC (e.g., *S. thermosulfidooxidans*). ORP, Fe^3+^ generation rate, and Fe^2+^ concentration represent the iron-oxidizing ability of iron-oxidizing bacteria in AMC (e.g., *L. ferriphilum*). Among them, Fe^3+^ generation rate was calculated through the change in the concentration of Fe^3+^ within a period of time.

Figure 2A shows that the pH of the leaching solution decreased gradually during the bioleaching process. The magnitude of the pH dropped in the leaching solution also decreased with the increase of pulp density of LiCoO_2_. AMC would gradually adapt to the changes of the bioleaching environment. At the same time, their acid production capacity would also gradually increase during the bioleaching process. However, the increase of pulp density of LiCoO_2_ would inhibit the acid production capacity of AMC.

The ORP of the leaching solution represented the iron-oxidative activity of AMC as shown in Figure 2B. The increase of ORP during the bioleaching process demonstrated the increase of iron-oxidative activity of AMC. However, the ORP decreased with the increase of pulp density, which meant that the iron-oxidizing ability of AMC could be suppressed due to the increase of pulp density of LiCoO_2_. The Fe^3+^ generation rate of the leaching solution as shown in Figure 2C also reflected the iron-oxidizing activity of AMC. When the slurry density of LiCoO_2_ reached 5.0%, the Fe^3+^ generation rate was lower than zero, which meant that the iron-oxidizing bacteria in AMC completely lost their iron-oxidizing ability.

In addition, the change of Fe^2+^ concentration shown in Figure 2D represented the energy-using ability of the iron-oxidizing bacteria in AMC from pyrite. Normally, when AMC quickly oxidized Fe^2+^ to Fe^3+^ to get energy, the Fe^2+^ concentration in the slurry would become smaller, while Fe^3+^ concentration would increase. However, Fe^2+^ concentration increased with time at 5% pulp density, which means that the AMC’s iron oxidation ability was reduced under a high pulp density.

Therefore, the iron oxidizing and sulfur-oxidizing abilities of AMC could be suppressed due to the high pulp density. Meanwhile, the bioleaching efficiencies of lithium and cobalt from LiCoO_2_ were also be impacted by the high pulp density, as shown in Figures 2E,F. The bioleaching efficiencies of lithium and cobalt at 4.0% pulp density within the first 24 h were 94.8 and 97.8%, respectively. The pH, ORP, Fe^3+^ generation rate, and Fe^2+^ concentration was 1.19, 533 V, 1.1 g⋅L^–1^⋅day^–1^, and 0.4 g⋅L^–1^ on the 5th day, respectively, indicating that AMC still maintained relatively high sulfur-oxidizing and iron-oxidizing activities at pulp density of 4.0%. However, the bioleaching efficiency of lithium and cobalt decreased to 87.6 and 87.0%, respectively, together with a decrease of bio-oxidative activity of AMC under 5.0% pulp density. All the data suggested that the bioleaching efficiencies of lithium and cobalt from LiCoO_2_ were adversely impacted by the pulp density of LiCoO_2_.

According to the bio-oxidizing mechanism of iron-oxidizing bacteria (Herve et al., 2008), electrons produced from bio-oxidation of iron-oxidizing bacteria, e.g., *L. ferriphilum* and *S. thermosulfidooxidans*, are partially transferred to NAD^+^ to provide energy for bacterial growth. Meanwhile, protons produced through bio-oxidation of elemental sulfur by sulfur-oxidizing bacteria, e.g., *S. thermosulfidooxidans*, are used for bioleaching of lithium and cobalt from LiCoO_2_. The mechanism of bioleaching LiCoO_2_ is proposed that Li^+^ is released merely through bio-acid leaching. However, Co^2+^ is leached through both bio-acid leaching and chemical reduction reaction between Co^3+^ and Fe^2+^. The trivalent cobalt in LiCoO_2_ competes with bacteria for electrons from ferrous ions and is reduced to divalent cobalt (Xin et al., 2016). That is why the concentrations of both Li^+^ and Co^2+^ increased with the increase of pulp density of LiCoO_2_. The impact of high concentrations of Li^+^ and Co^2+^ on the bio-oxidative activity of AMC became more and more severe, which would lead to the metal ion stress in AMC. This also explained why the bio-oxidative activity of AMC and bioleaching efficiencies of lithium and cobalt under 5.0% pulp density of LiCoO_2_ decreased within the first 24 h compared with others as shown in Figure 2C. Thus, it was important to investigate the impact of Li^+^ and Co^2+^ concentrations on the bioleaching of LiCoO_2_ of AMC under a high pulp density.

### Impact of Initial Lithium and Cobalt Ion Concentrations on the Bio-Oxidative Activity of AMC

Several researchers have proved that there exist different tolerances of AMC for various metal ions such as Mo^6+^ and V^4+^ (Pradhan et al., 2009), Cu^2+^ (Navarro et al., 2013), and Zn^2+^ (Chen et al., 2004; Xin et al., 2012) based on diverse mechanisms (Wu et al., 2014). However, few have reported about the tolerance of AMC to high concentrations of Li^+^ and Co^2+^.

**FIGURE 3:**
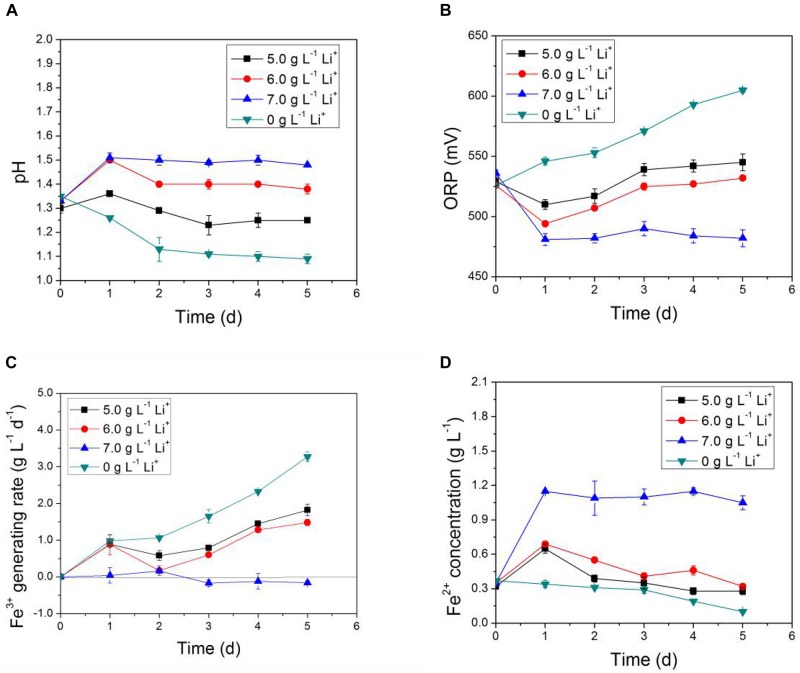
Time-courses for **(A)** pH, **(B)** ORP, **(C)** Fe^3+^ generation rate, and **(D)** Fe^2+^concentration under simulated bioleaching system of different Li^+^ concentrations.

**FIGURE 4:**
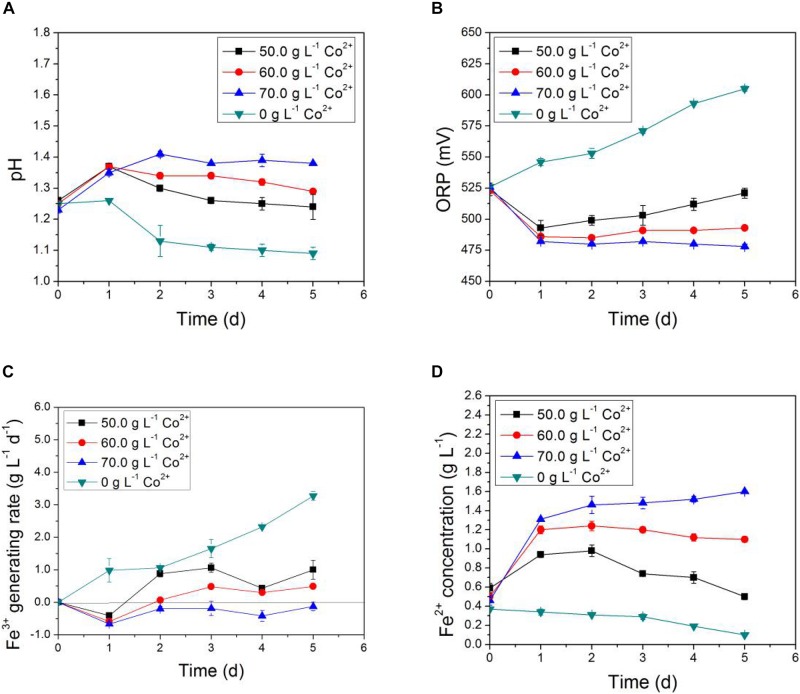
Time-courses for **(A)** pH, **(B)** ORP, **(C)** Fe^3+^ generation rate, and **(D)** Fe^2+^concentration in simulated bioleaching system of different Co^2+^ concentrations.

In Figures 3, 4, the impact of Li^+^ concentration on the bio-oxidative activity of AMC was similar to that of Co^2+^ in the simulated bioleaching systems that added only one of the two. It was obvious that the acid-producing and iron-oxidizing abilities of AMC decreased with the increase of the concentration of either Li^+^ or Co^2+^. In Figures 3A, 4A, the pH of the leaching solution decreased gradually with time. This means that Li^+^ or Co^2+^ had a certain inhibition effect on the acid-producing ability of the sulfur-oxidizing bacteria in AMC. The ORP and Fe^3+^ generation rate decreased with the increase of initial Li^+^ or Co^2+^ concentrations as shown in Figures 3B,C, 4B,C, which indicate that there was an inhibition effect of Li^+^ or Co^2+^ on the iron-oxidizing ability of bacteria in AMC. Therefore, in Figures 3D, 4D, there was an accumulation of Fe^2+^ because it could not be oxidized fast enough. When the concentration of Li^+^ was 7.0 g⋅L^–1^ or the concentration for Co^2+^ was 70.0 g⋅L^–1^, AMC lost most of its ability to oxidize sulfur and iron. The corresponding pulp densities of LiCoO_2_ were 10.0 and 12.0%, respectively. Thus, the toxicity of Li^+^ or Co^2+^ was not the direct cause of the loss of bio-oxidative activity of AMC under pulp density of 5.0% LiCoO_2_.

### Impact of Coexisting Li^+^ and Co^2+^ on Bio-Oxidative Activity of AMC

**FIGURE 5:**
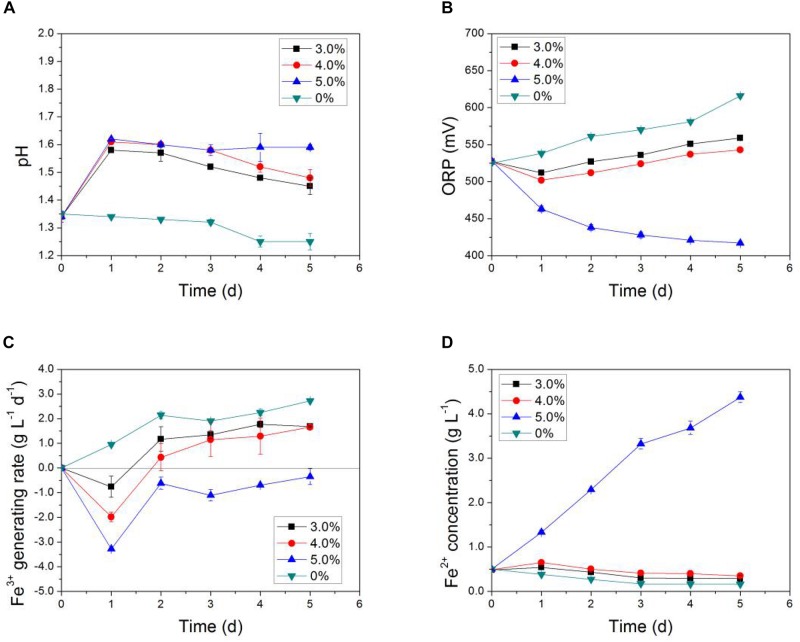
Time-courses for **(A)** pH, **(B)** ORP, **(C)** Fe^3+^ generation rate, and **(D)** Fe^2+^concentration in simulated bioleaching system with varied pulp density of LiCoO_2_.

Synergistic effects of Li^+^ and Co^2+^ present at the same time on the bio-activity of AMC were investigated using the simulated bioleaching systems of LiCoO_2_ with the results shown in Figure 5. In Figure 5, the pH of the simulated bioleaching system of 3.0 and 4.0% pulp densities increased in the first day and then decreased, which indicated that the availability of the acids produced by the AMC decreased first and then began to recover after 1 day of inhibition. However, the pH of the simulated bioleaching system of 5.0% pulp density did not show any decrease. As to the iron-oxidizing ability, the ORP value and Fe^3+^ generation rate of the simulated bioleaching system at 5.0% pulp density continued to decrease with time. This meant that iron-oxidizing bacteria in AMC, like *L. ferriphilum* and *S. thermosulfidooxidans*, were not as active as before performing oxidation of iron to obtain energy.

In general, a synergistic effect between Li^+^ and Co^2+^ on the bio-oxidation activity of AMC was observed. Data here suggest that the toxicity of both Li^+^ and Co^2+^ together was the direct cause of the bioleaching activity loss of AMC under pulp density of 5.0% of LiCoO_2_. This conclusion is different from the bioleaching result of spent Zn–Mn batteries (Xin et al., 2012), in which it is not the toxicity of Zn^2+^ and Mn^2+^ that led to the decreased bioleaching efficiency of Zn and Mn. The deteriorated environmental conditions including pH and ORP at elevated pulp densities are responsible for the decreased bioleaching efficiency. This difference might be mainly due to the different compositions of metals between LiCoO_2_ and Zn–Mn battery wastes, as well as the tolerance of different microorganisms to different metals. Therefore, the impact of coexisting Li^+^ and Co^2+^ on the bioleaching activity of AMC should be considered in practical bioleaching operations.

### Intracellular ROS Content of AMC With Coexisting Li^+^ and Co^2+^

As discussed above, the toxicity of both Li^+^ and Co^2+^ together was the direct cause of the bioleaching activity loss of AMC under a 5.0% pulp density of LiCoO_2_. However, the specific mechanism for the effect of coexisting Li^+^ and Co^2+^ on the bioleaching activity of AMC is still unclear. The following hypothesis was proposed: the oxidative stress in AMC cells would occur due to the coexistence of Li^+^ and Co^2+^. Moreover, excessive intracellular ROS induced by metal ions would also affect the bioleaching activity of AMC. Thus, this study focused on the change of intracellular ROS content induced by coexisting Li^+^ and Co^2+^ in the simulated bioleaching system.

**FIGURE 6:**
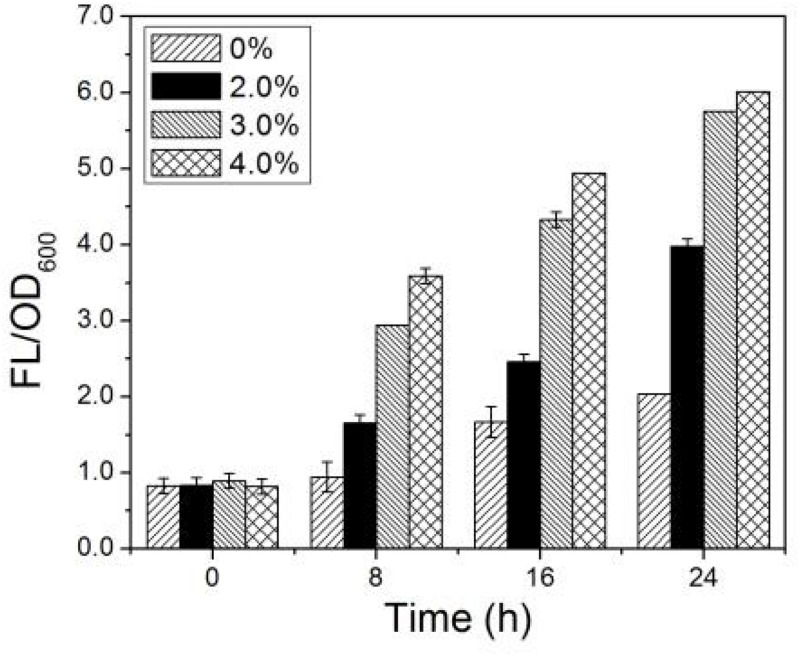
Time-courses for FL/OD_600_ for simulated bioleaching system with varied pulp density.

The changes of intracellular ROS content in AMC as a function of time in the simulated bioleaching system with 2.0, 3.0, and 4.0% pulp densities of LiCoO_2_ are illustrated in Figure 6. In Figure 6, the intracellular ROS content of AMC increased with the metal ion concentrations and time. Large changes were observed for the intracellular ROS content with pulp density, in the following order: ROS (4.0%) > ROS (3.0%) > ROS (2.0%). The higher concentrations of coexisting Li^+^ and Co^2+^, the more intracellular ROS accumulated in AMC. In the simulated bioleaching system with 4.0% pulp density, the intracellular ROS content in AMC increased from 0.8 to 6.0 within 24 h, while that of the control just increased from 0.8 to 2.0. Thus, it was possible that the biological activity and bioleaching ability of AMC for lithium and cobalt could be affected seriously by the vast increase in the intracellular ROS content.

It is well known that oxidative stress can disrupt the balance between the generation and elimination of intracellular ROS and dramatically increase the intracellular ROS content (Liang and Zhou, 2007), which will further result in the peroxidation of lipid membrane accumulation of MDA (Dat et al., 2000) damage of DNA and the reduction of the caspase-3 enzyme activity (Kim et al., 1995). However, it is also found that there exist intracellular antioxidant enzyme systems (Rodrigues et al., 2010) and non-enzyme antioxidant components (Xu et al., 2016) in microorganisms to maintain the balance of ROS in cells. It is reported that the thiol/disulfide system plays a crucial role in red ox protection in *L. ferriphilum* (Javiera et al., 2012). Previous studies have shown that the intracellular ROS content could be regulated by exogenous chemicals such as cobalamin (Chen et al., 2016; Ferrer et al., 2016). For the bioleaching system of WLIBs in this study, further studies were needed to confirm whether there exist some similar exogenous antioxidant chemicals to regulate the intracellular ROS levels in AMC.

### Impact of Exogenous GSH on Bio-Oxidation Activity, Intracellular ROS Content, Activities of Intracellular ROS Scavenging Enzymes

Glutathione is a small molecular (Muller, 2011) which can help microorganisms to alleviate ionic, osmotic, and oxidative stresses induced by salinity (Liu et al., 2013). It can eliminate intracellular ROS (Deleve and Kaplowitz, 1991) to maintain a normal red ox level in cells (Hayat et al., 2012). The effects of exogenous GSH on improving growth activity of bacteria have been demonstrated in many papers (Goswami et al., 2006; Wang et al., 2016), but no report was found to illustrate the impact of exogenous GSH on the bioleaching of LiCoO_2_ by AMC. In this paper, exogenous GSH was used as an ROS scavenger for AMC during the bioleaching of LiCoO_2_. The effect of the addition of 0.3 g⋅L^–1^ GSH to the 9K medium on the bio-oxidative activity, intracellular ROS content, the activities of ROS scavenging enzymes of AMC in the simulated bioleaching system with 4.0% of LiCoO_2_ were investigated experimentally.

**FIGURE 7:**
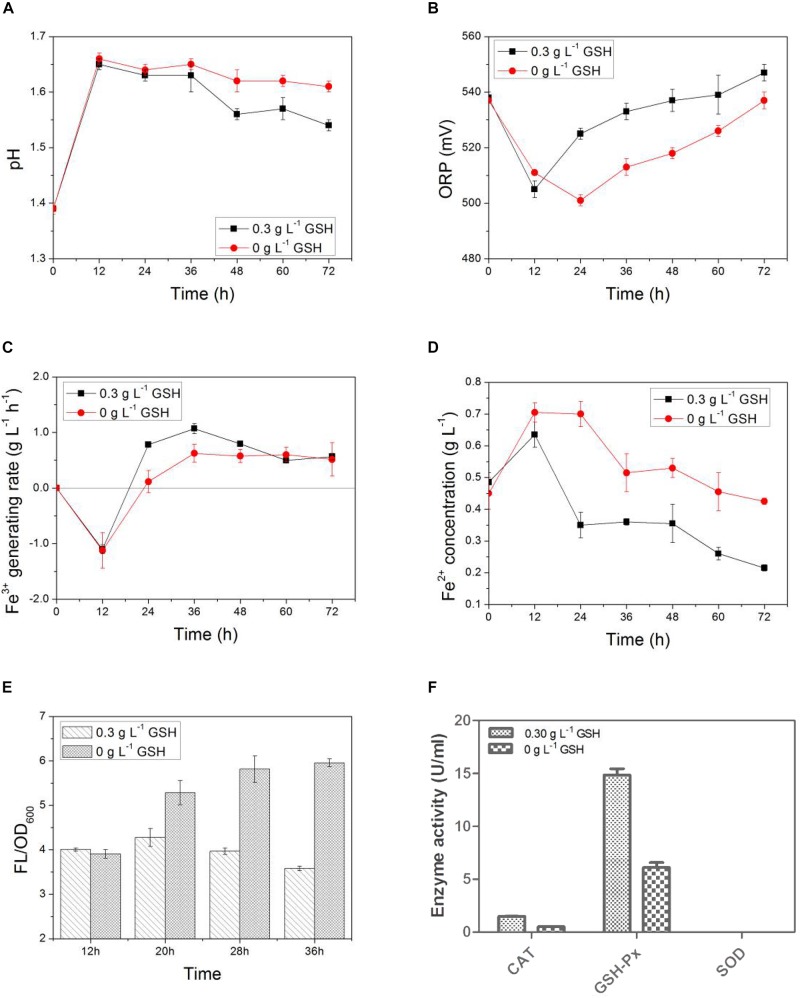
Time-courses for **(A)** pH, **(B)** ORP, **(C)** Fe^3+^ generation rate, **(D)** Fe^2+^concentration, **(E)** ROS content, and **(F)** activities of intracellular ROS scavenging enzyme by exogenous GSH for simulated bioleaching system with 4.0% (w⋅v^–1^) pulp density of LiCoO_2_.

In Figure 6, exogenous GSH was added at the 12th hour after the start of the bioleaching process. As shown in Figure 7A that the pH values of both the GSH-containing group and GSH-free group decreased with time, but the magnitude of the pH drop of the former was greater than that of the later. This suggests that exogenous GSH could intensify the sulfur-oxidizing ability of AMC to produce acid. Similarly, the ORP values and Fe^3+^ generation rates of both the GSH-containing group and the GSH-free group showed the same trend as shown in Figures 7B,C. The Fe^3+^ generation rate of the GSH-containing group at the 24th hour increased to 0.8 g⋅L^–1^⋅d^–1^, which was sixfolds higher than that of the GSH-free group. It was demonstrated that exogenous GSH could enhance the iron-oxidizing ability of AMC. Therefore, more Fe^2+^ accumulated in the GSH-free group than that in GSH-containing group as shown in Figure 7D.

The intracellular ROS content of AMC in the GSH-free group increased with time because of the synergistic effect of Li^+^ and Co^2+^ as shown in Figure 7E, which increased from 4.0 at 12th hour to 6.0 at 36th hour. Because exogenous GSH could significantly inhibit the ROS increase in AMC, the ROS content of GSH-containing group decreased from 4.0 to 3.6. There might be causal relationship between ROS content and bio-oxidation activity. Thus, the activities of ROS scavenging enzymes of AMC, such as GSH-Px, CAT, and SOD, were evaluated below.

In Figure 7F, the enzyme activities of GSH-Px, CAT in the GSH-containing group were 1.4- and 2.0-folds higher than those in the GSH-free group, respectively. It can be speculated that GSH intensified the activities of GSH-Px and CAT to scavenge the excessive ROS. It was possible that there existed an intracellular antioxidant system in AMC. However, no SOD activity was detected in both the GSH-containing group and the GSH-free group, which indicated that SOD did not play a significant antioxidant role in the intracellular antioxidant system of AMC during the bioleaching of LiCoO_2_.

Although researchers have pointed out that some of the acid-producing microorganisms, for example, *L. ferrooxidans*, have no ROS scavenging enzymes systems (e.g., SOD and CAT) or proteins belonging to a GSH system to maintain an intracellular red ox equilibrium (Ferrer et al., 2016). A predicted gene encoding OxyR, a transcription factor that regulates the response to red ox stress was found in *L. ferriphilum* but not in *A. ferrooxidans*, suggesting that there are differences among the molecular mechanisms for the regulation of oxidative stress response from various microorganism (Cortés et al., 2011).Javiera et al. (2012) suggested that *L. ferriphilum* is mainly protected by the thioredoxin-based thiol/disulfide system to survive under extreme oxidative environments. It was found in this study that exogenous GSH increased the activities of ROS scavenging enzymes in AMC to scavenge the excessive intracellular ROS content. The bio-oxidative activity of AMC also increased.

There were three possible reasons to explain the functions of exogenous GSH here. First, the addition of GSH could eliminate the intracellular ROS and inhibit the further production of ROS, while also increasing the activity of intracellular antioxidant enzymes (Hasan et al., 2016). The expression of antioxidant genes encoding enzymes and certain regulators of cell senescence and apoptosis will also be upregulated after the GSH addition (Chen et al., 2016). For example, it is possible that the function of the thioredoxin based thiol/disulfide system may be enhanced by exogenous GSH in the red ox protection of *L. ferriphilum* under a high pulp density of LiCoO_2_. Second, exogenous GSH can stimulate the production of non-enzymatic small molecule antioxidants such as AsA, GSH, etc. (Cristiano et al., 2016). Third, exogenous GSH can chelate metal ions to mitigate the oxidative stress induced by metal ions in AMC (Wang et al., 2018). In summary, the regulation of exogenous GSH on the bioleaching antioxidant activity of AMC was coordinated by multiple response pathways. These would be investigated carefully in the near future.

### Bio Film Under the Condition of Stress by Li Ion and Cobalt Ion

It has been reported that ROS is associated with the reduction in the amount of bio film formation (Suo et al., 2012). It was also suggested that the formation of bio films and the secretion of extracellular polysaccharides are also part of the ROS defense strategies of *L. ferriphilum* (Bellenberg et al., 2014). However, there is no public report on whether ROS induced by lithium ion and cobalt ion has a certain effect on the formation of an acidophilic bio film.

**FIGURE 8:**
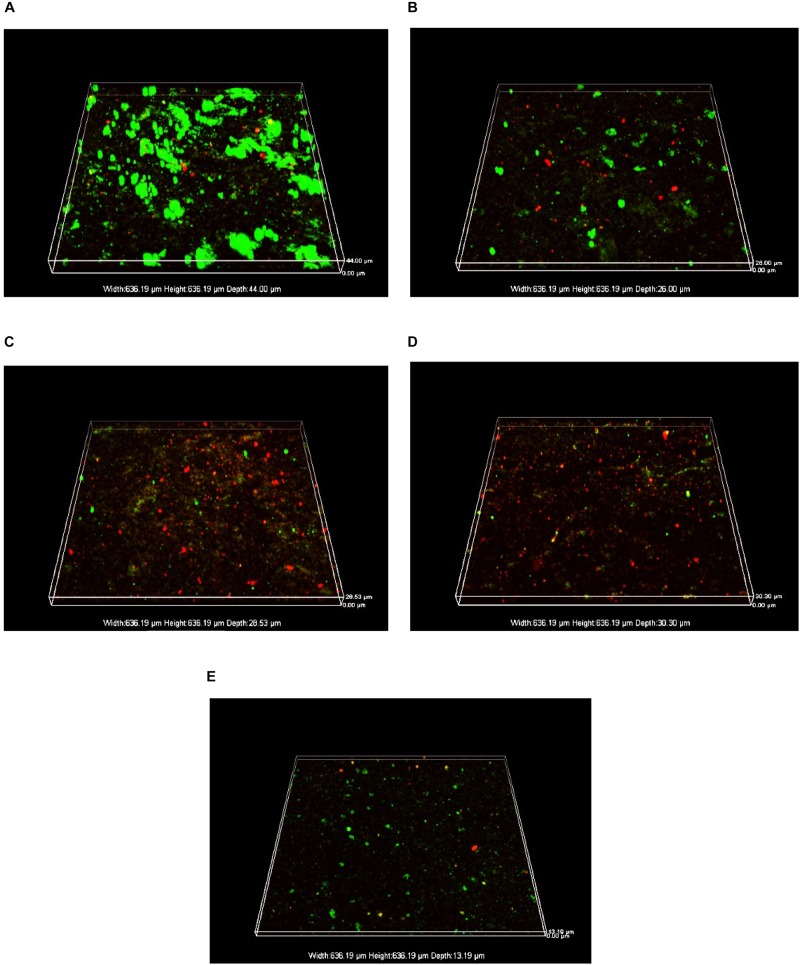
CLSM images of biofilms after 7-day incubation on the surfaces of pyrite coupons: **(A)** no lithium and cobalt ions (control), **(B)** 2.84 g⋅L^–1^ Li^+^, **(C)** 24.08 g⋅L^–1^ Co^2+^, **(D)** 2.84 g⋅L^–1^ Li^+^ + 24.08 g⋅L^–1^ Co^2+^, and **(E)** 2.84 g⋅L^–1^ Li^+^ + 24.08 g⋅L^–1^ Co^2+^ + 0.30 g⋅L^–1^ GSH.

The acidophilic bio film on the surface of the pyrite coupon after 7 days of incubation was observed under CLSM for conditions with lithium ion, cobalt ion, as well as lithium ions and cobalt ion coexisting, respectively. Figure 8A shows that in the absence of lithium and cobalt ions (control group), live sessile bacteria (green dots) were dense in clusters with hardly any dead sessile cells (red dots), suggesting that the acidophilic microorganisms grew well on the pyrite coupon.

In comparison, in the presence of 2.84 g⋅L^–1^ Li^+^ (Figure 8B), live sessile cells were sparse and dead sessile cells were obviously present. This indicates that under the stress of lithium ion, the bacterial activity was inhibited, and the biofilm-forming ability was weakened. A similar phenomenon also occurred in the presence of 24.08 g⋅L^–1^ Co^2+^ as shown in Figure 8C.

When both lithium and cobalt ions were present, there were fewer live and dead sessile cells on the pyrite coupon as shown in Figure 8D. This means that cells either did not attach or the attached sessile cells were mostly killed. This indicates that coexisting lithium and cobalt ions were more harmful to sessile cells than one of them alone because ROS induced by metal ions damaged the bio film formation more seriously in the former case. However, when GSH was added under the metal ion stress as shown in Figure 8E, the live sessile cell count rebounded and there were few dead cells, indicating that the metal ion stress was alleviated to some extent.

The sessile cell counts for different conditions were carried out experimentally. The sessile cell count was 7.74 × 10^6^ cells⋅cm^–2^ in the control group, and 1.86 × 10^6^ cells⋅cm^–2^ in lithium ion and cobalt ion coexisting group, suggesting a fourfold reduction due to the effect of metal ion stress. When GSH was added to the lithium–cobalt ions coexisting group, the sessile cell count rebounded to 2.48 × 10^6^ cells⋅cm^–2^, indicating that the effect of metal ions stress on the bio film was partially alleviated. These results are consistent with the CLSM images.

Recent studies have shown that the sessile bacteria can secrete exopolymeric substances (EPS), such as proteins, DNA, and polysaccharides, to embed sessile cells to withstand adverse external conditions. Moreover, there is the exchange and transmission of mass, energy, and information among different species in the bio film (Vera et al., 2018), which makes the bio film an ecosystem different from the bulk fluid of the leaching solution. It is in this micro-ecological environment underneath a bio film, there are a lower pH and higher iron ion concentrations due to a higher volumetric sessile cell density than the planktonic cell density, so that acidophilic microorganisms can maintain a strong bioleaching activity. Eventually, it can lead to more efficient leaching of lithium and cobalt ions from LiCoO_2_. Under the condition of a high concentration of metal ions, metal ion stress induced oxidative stress on acidophilic microorganisms, which reduces the ability of bacteria to secrete EPS and thus inhibiting the formation of bio film. Most acidophilic bacterial cells were present as planktonic bacteria. Therefore, the lithium and cobalt recoveries from LiCoO_2_ were largely reduced. This should be the main reason for the decrease in bioleaching efficiency at a high pulp density of LiCoO_2_ slurry.

### Improving Pulp Density of Bioleaching of LiCoO_2_ With Exogenous GSH

**FIGURE 9:**
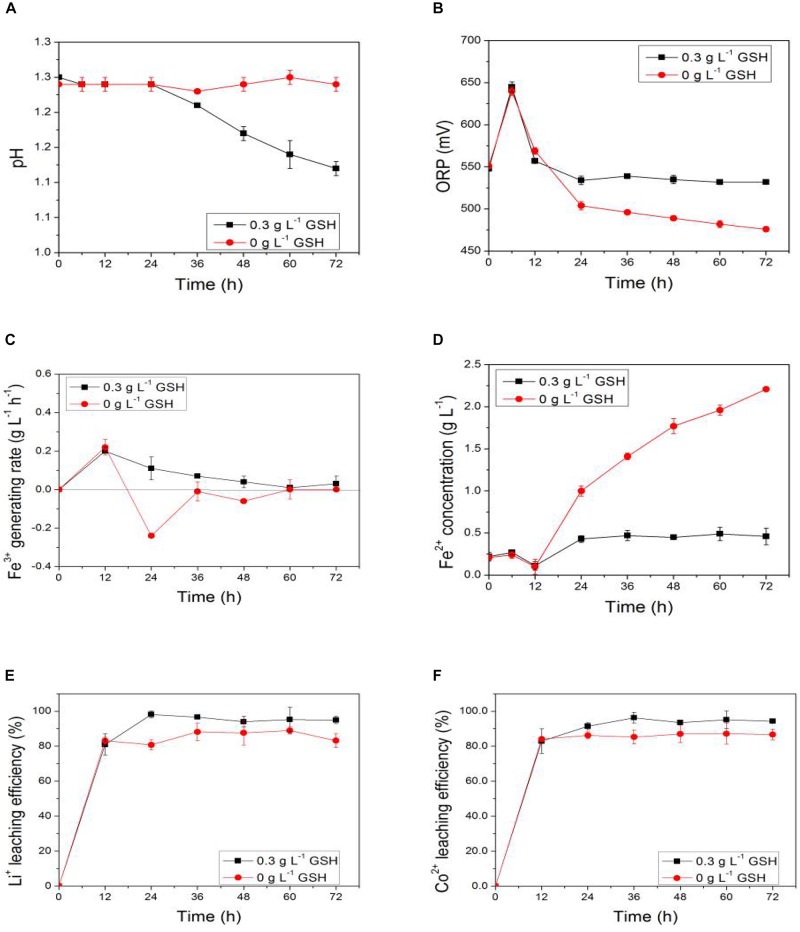
Time-courses for **(A)** pH, **(B)** ORP, **(C)** Fe^3+^ generation rate, **(D)** Fe^2+^concentration, **(E)** bioleaching efficiency of lithium, and **(F)** bioleaching efficiency of cobalt under pulp density of 5.0% with and without exogenous GSH.

Bioleaching of lithium and cobalt under 5.0% pulp density of LiCoO_2_ was carried out with the addition of 0.3 g⋅L^–1^ of GSH at the beginning. The changes of ORP value, Fe^3+^ generation rate, Fe^2+^ concentration, and the leaching efficiencies of lithium and cobalt from LiCoO_2_ with time are shown in Figure 9. In Figure 9A, it is obvious that the pH of GSH group decreased gradually with time after the addition of GSH. This suggests that the sulfur-oxidizing ability of AMC was intensified by exogenous GSH. In Figure 9B, the red ox potential of the leaching solution was significantly increased by the exogenous GSH. The Fe^2+^ concentration remained stable at a relatively low level compared to the GSH-free group. Figure 9C shows that the Fe^3+^ generation rate in GSH group was also significantly increased and remained positive. Thus, Fe^2+^ concentration would not increase as shown in Figure 9D, which means that the addition of GSH for the bioleaching of LiCoO_2_ could effectively intensified the iron-oxidizing ability of AMC.

Figures 9E,F show that the sulfur-oxidizing and iron-oxidizing abilities of AMC under 5.0% pulp density of LiCoO_2_ were completely inhibited in the GSH-free group. Furthermore, the bioleaching efficiencies of both lithium and cobalt from LiCoO_2_ were higher in the GSH groups than those in the GSH-free group. For example, the bioleaching efficiency of lithium of the former increased to 98.1% in 24 h at 5.0% pulp density of LiCoO_2_, and that of cobalt increased to 96.3% in 36 h. Thus, the addition of GSH not only improved the activity of bio-oxidation, but also increased the bioleaching efficiencies. This means exogenous GSH is a good way to improve the bioleaching under a high pulp density.

Accordingly, the mechanism of the effect of a high pulp density on the bioleaching process could be proposed. There existed an effect of metal-ion stress on the bio-oxidative activity of AMC during the bioleaching of LiCoO_2_. The Li^+^ and Co^2+^ accumulated in the leaching solution were the direct cause for the decrease in lithium and cobalt recovery yields under the high pulp density, which would lead to the decrease of activities of ROS scavenging enzymes and the increase of intracellular ROS content in AMC. The oxidative stress induced by metal ions in AMC then occurred. Finally, the bio-oxidative activity and bioleaching efficiencies of lithium and cobalt from LiCoO_2_ by AMC would gradually decrease due to the damage by intracellular ROS. In other words, the increase of intracellular ROS content induced by the synergistic effect of Li^+^ and Co^2+^ could further lead to failure of intracellular ROS homeostasis, which was one of the key factors for the influence of the high pulp density, leaching time and leaching efficiency of lithium and cobalt on the bioleaching of LiCoO_2_.

Using exogenous GSH could effectively regulate the activities of intracellular ROS scavenging enzymes and reduce the excess intracellular ROS content in AMC. Thus, the bioleaching efficiencies under the high pulp density could be improved by this strategy. Table 1 compares this work with published data. The bioleaching efficiencies of 98.1% for lithium and 96.3% for cobalt were obtained within 1.5 days under the pulp density of 5.0% of LiCoO_2_ in this study. Compared with the published data, a shorter leaching time, higher leaching efficiencies, and a higher pulp density for the bioleaching of LiCoO_2_ were achieved in this work. This was mainly attributed to the regulation of intracellular ROS content in AMC and the formation of bio film of AMC in the bioleaching of LiCoO_2_.

**TABLE 1:** Comparison of this study with published data on the bioleaching of LiCoO_2_.

**Material**	**Microorganism**	**Efficiency**	**Time (days)**	**References**
0.5% LiCoO_2_	*A. ferrooxidans*	Li 10.0%; Co 65.0%	20	Mishra et al., 2008
1.0% ASP material	*A. thiooxidans*	Li 80.0%; Co 10.0%	5	Xin et al., 2016
1.0% LiCoO_2_	*A. thiooxidans* and *L. ferrooxidans*	Li 80.0%; Co 90.0%	5	Xin et al., 2009
1.0% LiCoO_2_	*A. ferrooxidans*	Co 98.4%	7	Zeng et al., 2013
1.0% LiCoO_2_	*A. ferrooxidans*	Co 99.0%	6	Zeng et al., 2011
1.0% cellular battery	*Aspergillus niger*	Li 95.0%; Co 45.0%	30	Horeh et al., 2016
4.0% LiCoO_2_	*Alicyclobacillus* spp. and *Sulfobacillus* spp.	Li 37.0%; Co 10.0%	12	Niu et al., 2014
5.0% LiCoO_2_	AMC	Li 98.1%; Co 96.3%	1.5	This work

Despite the abundant data presented in this work, the evidence at the molecular-genetic level of AMC about the effects of pulp density on the biooxidation activities and bioleaching efficiencies are desired. Future studies will likely employ metagenomics and transcriptomics.

## Conclusion

The mechanism to explain the effects of a high pulp density on the bioleaching of LiCoO_2_ was proposed. The oxidative stress caused by the synergistic effect between coexisting Li^+^ and Co^2+^ under the high pulp density would lead to the increase of intracellular ROS content and the disruption of intracellular ROS homeostasis. The sessile growth of the AMC on the pyrite surface was inhibited under the condition of coexisting lithium and cobalt ions, making bio film formation difficult, which further led to the decrease of bio-oxidative activity of AMC and the bioleaching efficiencies of lithium and cobalt from LiCoO_2_. However, the addition of GSH could reduce the amount of intracellular ROS induced by the metal ion stress and also improve the bio film forming ability of AMC and the bioleaching activity of LiCO_2_. High bioleaching efficiencies of lithium of 98.1%, and cobalt of 96.3% under the 5% pulp density of LiCoO_2_ were obtained in this paper with the addition of 0.3 g⋅L^–1^ GSH.

## Data Availability Statement

The datasets generated for this study are available on request to the corresponding author.

## Author Contributions

XL, WW, HL, XZ, TG, MZ, and WT designed the experiments. XL, HL, and WW carried out the experiments. XL, XZ, and TG analyzed the experimental results. XL, HL, XZ, and TG wrote the manuscript. All the authors edited the manuscript.

## Conflict of Interest

The authors declare that the research was conducted in the absence of any commercial or financial relationships that could be construed as a potential conflict of interest.
